# Water quality and Inuit health: an examination of drinking water consumption, perceptions, and contamination in Rigolet, Canada

**DOI:** 10.1080/22423982.2017.1335149

**Published:** 2017-06-14

**Authors:** Carlee Wright

**Affiliations:** ^a^ Department of Population Medicine, University of Guelph, Guelph, Canada; ^b^ MSc Population Medicine

## Abstract

Canadian Inuit have often reported concerns about the quality of their municipal drinking water; research has also shown that some Inuit communities experience some of the highest incidence rates of self-reported acute gastrointestinal illness (AGI) in Canada and globally. The goal of this thesis research was to investigate drinking water perceptions and consumption patterns, as well as water contamination and potential associations with AGI in the Inuit community of Rigolet, Canada. Three census cross-sectional surveys captured data on AGI, drinking water, and water storage (2012–2014); additionally, bacterial contamination of household drinking water was assessed alongside the 2014 survey. Concerns regarding the taste, smell, and colour of tap water were associated with lower odds of consuming tap water. The use of transfer devices (i.e. small bowls or measuring cups) was associated with household water contamination; while no water-related risk factors for AGI were identified, incidence of AGI was high compared with southern Canada. This thesis research provides a valuable contribution to the limited literature assessing drinking water and health in the Arctic. Ultimately, this work is intended to inform safe water management practices, as well as contextually appropriate drinking water interventions, risk assessments, and public health messaging in the Canadian Arctic.

**1.**
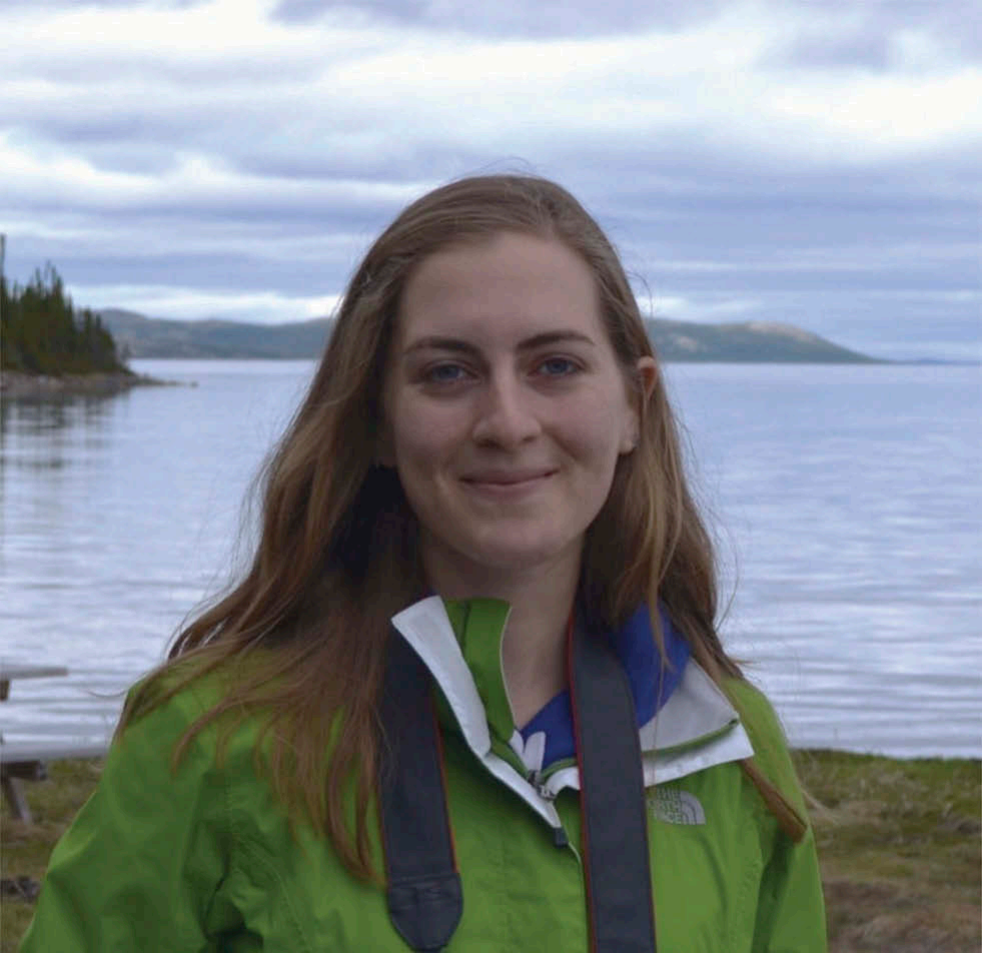
Carlee Wright (photo credit: Sherilee Harper).

